# Nonlinear Relationship Between Triglyceride‐to‐High‐Density Lipoprotein Cholesterol Ratio and Non‐Alcoholic Fatty Liver Disease: A Secondary Retrospective Analysis Based on a Japanese Longitudinal Study

**DOI:** 10.1002/jcla.70107

**Published:** 2025-09-25

**Authors:** Lingde Shen, Yuanfang Lin, Weifeng Chen, Dan Zhou, Hui Peng

**Affiliations:** ^1^ Department of Tuina Shenzhen Traditional Chinese Medicine Hospital (The Fourth Clinical Medical College of Guangzhou University of Chinese Medicine) Shenzhen China; ^2^ Department of General Practice Shenzhen Second People's Hospital (The First Affiliated Hospital of Shenzhen University) Shenzhen China

**Keywords:** high‐density lipoprotein cholesterol, non‐alcoholic fatty liver disease, nonlinearity, triglyceride, triglyceride to high‐density lipoprotein cholesterol ratio

## Abstract

**Background:**

The purpose of this research is to investigate the particular connection between the triglyceride to high‐density lipoprotein cholesterol (TG/HDL‐C) ratio and non‐alcoholic fatty liver disease (NAFLD) to offer a more precise foundation for evaluating NAFLD risk.

**Methods:**

This study involves a secondary analysis of a retrospective cohort study conducted from 2004 to 2015 in a Japanese population, which included 14,106 participants. The TG/HDL‐C ratio was determined by the levels of triglycerides (TG) and high‐density lipoprotein cholesterol (HDL‐C). Participants were grouped according to the quartiles of TG/HDL‐C. We analyzed the relationship between TG/HDL‐C and NAFLD using Cox proportional hazards regression, smooth curve fitting, and sensitivity analysis.

**Results:**

The average age of the study participants was 43.51 ± 8.89 years, with 7275 (51.57%) being male. After considering potential confounding factors, the study found a positive correlation between TG/HDL‐C and NAFLD (OR: 1.37, 95% CI: 1.31–1.43, *p* < 0.001). Moreover, a nonlinear relationship between TG/HDL‐C and NAFLD was found, with a turning point at 1.42. The odds ratio (OR) on either side of this inflection point were 3.71 (95% CI: 2.87–4.79) on the left and 1.23 (95% CI: 1.17–1.29) on the right, indicating a stronger correlation when TG/HDL‐C is below 1.42, particularly in younger individuals, females, and those with a BMI under 25 kg/m^2^.

**Conclusion:**

The TG/HDL‐C index shows a nonlinear positive correlation with NAFLD risk, particularly when the TG/HDL‐C ratio is below 1.42, with a stronger association observed in younger individuals, females, and lower‐BMI populations.

## Graphical abstract

This study reveals a nonlinear positive correlation between the TG/HDL‐C ratio and the risk of NAFLD. The association is particularly pronounced when the ratio is below 1.42.

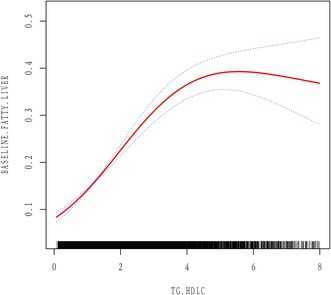

**Abbreviations**
ALT: alanine aminotransferase
AST: aspartate aminotransferase
BMI: body mass index
CI: confidence interval
DBP: diastolic blood pressure
FFAs: free fatty acids
FPG: fasting plasma glucose
GGT: gamma‐glutamyl transferase
HbA1c: glycosylated hemoglobin A1c
HDL‐C: high‐density lipoprotein cholesterol
NAFLD: non‐alcoholic fatty liver disease
NASH: non‐alcoholic steatohepatitis
OR: odds ratio
SBP: systolic blood pressure
TC: total cholesterol
TG: triglyceride
TG/HDL‐C: triglyceride‐to‐high‐density lipoprotein cholesterol ratio
WC: waist circumference

## 1 Introduction

In recent years, the ratio of triglycerides to high‐density lipoprotein cholesterol (TG/HDL‐C) has gained recognition as a significant marker for evaluating metabolic health [1, 2]. An elevated TG/HDL‐C ratio is often linked to insulin resistance, inflammatory responses, and lipid metabolism disorders, all of which are major pathogenic factors of non‐alcoholic fatty liver disease (NAFLD) [3, 4]. The mechanisms underlying NAFLD are complex, and its significance lies not only in its own health risks but also in the potential progression to more severe conditions, such as non‐alcoholic steatohepatitis (NASH), liver fibrosis, and even liver cancer [5, 6]. Research has shown that the equilibrium between TG and HDL‐C is crucial in the pathophysiology of NAFLD [7, 8]. High TG levels are often closely related to the accumulation of liver fat and insulin resistance, whereas HDL‐C is considered to have a protective role, alleviating inflammation and oxidative stress [9, 10]. Therefore, changes in the TG/HDL‐C ratio may reflect the dynamic state of these metabolic disturbances, providing a new perspective for assessing the risk of NAFLD [11, 12].

Although the TG/HDL‐C ratio is increasingly seen as valuable in NAFLD, more studies are required to grasp the intricate relationship between this ratio and NAFLD. Given that changes in TG and HDL‐C levels may be influenced by individual differences, environmental factors, and metabolic states, a single TG/HDL‐C ratio may be insufficient for a comprehensive assessment of NAFLD risk, necessitating further investigation of their relationship.

This research seeks to investigate the connection between the TG/HDL‐C ratio and NAFLD. By analyzing cross‐sectional sample data, we will evaluate the impact of changes in TG/HDL‐C ratio levels on NAFLD risk. We hope this research will provide evidence for a clearer insight into the association between TG/HDL‐C ratios and NAFLD, offering more accurate information for clinicians in assessing and managing NAFLD patients, ultimately facilitating the development of effective interventions and improving patient outcomes.

## 2 Methods

### 2.1 Data Source and Study Design

This study will analyze data from the NAGALA research group, whose design and objectives have been thoroughly described in previous literature [13]. Since 1994, this ongoing research initiative has been recruiting individuals from the general population who undergo health check‐ups at Murakami Memorial Hospital. This research primarily aims to examine health screening data to pinpoint chronic diseases and their risk factors, which greatly affect public health, offering crucial insights for creating chronic disease prevention strategies. The NAGALA study was ethically approved by the Murakami Memorial Hospital's ethics committee, and informed consent was secured from all participants (IRB2018‐09‐01). Professor Okamura has uploaded the research data to the Dryad database (https://doi.org/10.5061/dryad.8q0p192) [13] to facilitate further analysis by other researchers while adhering to the database's terms and conditions.

From 2004 to 2015, Murakami Memorial Hospital conducted a preliminary study involving 20,944 participants aged 18 and above, each of whom underwent at least two standard health check‐ups. This secondary analysis utilized data from the NAGALA cohort study, which initially enrolled 20,944 participants. The original study's exclusion criteria included the following: (1) individuals with a diagnosis of type 2 diabetes (*n* = 323) or fasting plasma glucose (FPG) levels higher than 6.1 mmol/L (*n* = 808); (2) individuals diagnosed with liver conditions, including hepatitis C or B infections (*n*=416); (3) individuals currently on medication (*n* = 2321); (4) individuals with alcohol use disorders (women consuming in excess of 40 g per day and men consuming over 60 g per day define this) (*n* = 739); and (5) participants with incomplete covariate data, including abdominal ultrasound, laboratory measurements, or information on physical activity and alcohol consumption (*n* = 863). Consequently, the resultant original dataset encompassed 15,464 participants.

For our NAFLD‐focused analysis, we implemented additional exclusion criteria to enhance the specificity of our investigation:

Stricter alcohol consumption limits: We excluded participants consuming > 210 g/week for men (*n* = 990) and > 140 g/week for women (*n* = 194). These thresholds are more conservative than the original study's criteria to ensure accurate NAFLD diagnosis by rigorously excluding potential alcohol‐related liver disease.

Extreme TG/HDL‐C values: Participants with TG/HDL‐C ratio > 8 (*n* = 165) were excluded, as these extreme values may represent measurement errors or severe metabolic disorders that could distort our analysis of the relationship between TG/HDL‐C and NAFLD.

Missing HDL‐C data: We excluded cases with unavailable HDL‐C measurements (*n* = 9) as this was essential for calculating our primary exposure variable (TG/HDL‐C ratio).

**FIGURE 1:**
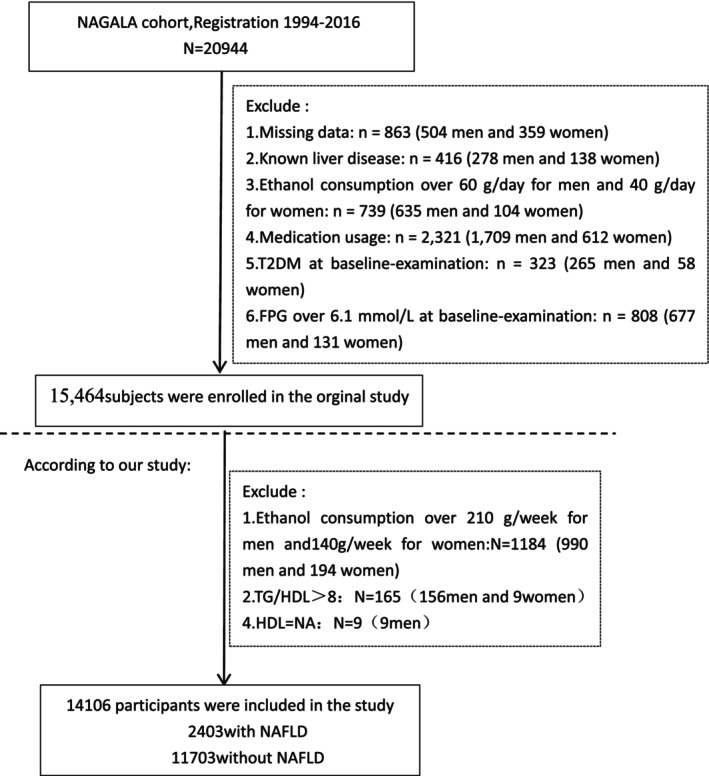
Flow diagram of subjects included in the study.

These additional exclusions resulted in our final analytical sample of 14,106 participants (2403 with NAFLD and 11,703 without NAFLD). The complete selection process is illustrated in Figure 1.

### 2.2 Definitions and Calculations

BMI is calculated as weight divided by height squared.

The TG/HDL‐C ratio is viewed as a continuous variable and is determined by dividing triglycerides by high‐density lipoprotein cholesterol.

### 2.3 Abdominal Ultrasound Diagnosis of NAFLD

Abdominal ultrasound is widely used to assess NAFLD. During this process, gastroenterologists perform a blinded evaluation of the ultrasound images, ensuring that participants' personal information is not obtained. The final diagnosis is determined by assessing four ultrasound parameters: liver echogenicity, hepatic‐renal echogenicity contrast, vascular blurring, and deep attenuation [14]. This ultrasound results scoring system has been applied to healthy Japanese adults. The AUC for NAFLD diagnosis was found to be 0.980, with a sensitivity of 91.7% (95% CI 87.0–95.1) and a specificity of 100%.

### 2.4 Other Variables

To collect details about participants' medical history and lifestyle factors like smoking, physical activity, and alcohol consumption, a self‐management questionnaire was employed. The database file includes the following variables [13]: age, sex, weight, waist circumference (WC), alanine aminotransferase (ALT), aspartate aminotransferase (AST), gamma‐glutamyl transferase (GGT), triglycerides (TG), total cholesterol (TC), high‐density lipoprotein cholesterol (HDL‐c), fasting plasma glucose (FPG), hemoglobin A1C (HbA1c), systolic blood pressure (SBP), diastolic blood pressure (DBP), exercise status, alcohol consumption status, and smoking status.

### 2.5 Statistical Analysis

We performed statistical analyses using Empower Stats (R) version 4.2 (http://www.empowerstats.com/cn/, X&Y Solutions Inc., Boston, MA, USA).

Quartiles were used to divide the TG/HDL‐C ratio into four categories: Q1 (0.07 < Q1 ≤ 0.69), Q2 (0.69 < Q2 ≤ 1.16), Q3 (1.17 < Q3 ≤ 2.08), and Q4 (2.08 < Q4 ≤ 7.99). Continuous variables that followed a normal distribution are presented as means with standard deviations, whereas those with a skewed distribution are represented as medians with interquartile ranges. Percentages are used to express categorical variables across various groups. Continuous variables were compared using one‐way ANOVA or Kruskal–Wallis tests, whereas chi‐square tests were used for categorical variables.

To evaluate how each variable affects the risk of NAFLD, a univariate Cox regression analysis was carried out. Additionally, the relationship between the TG/HDL‐C ratio and NAFLD was further examined using multivariate Cox regression analysis. To thoroughly examine the link between the TG/HDL‐C ratio and NAFLD, we employed models with varying levels of adjustment: the model 1 (no adjustments), model 2 (adjusted for sex and age), and model 3 (adjusted for sex, age, BMI, WC, weight, AST, ALT, GGT, SBP, DBP, HbA1c, FPG, TC, smoking, exercise, and drinking).

To explore the nonlinear relationship between the TG/HDL‐C ratio and NAFLD, we used Cox proportional hazards regression, along with cubic spline functions and smooth curve fitting. Our method for dealing with nonlinearity is based on an academic approach, initially using a recursive algorithm to locate the inflection point. Starting with an arbitrary initialization, this algorithm moves through a sequence of filtering and smoothing processes to accurately determine the inflection point. Subsequently, a two‐section Cox proportional hazards regression model was constructed to independently analyze the data on each side of the inflection point. With this comprehensive analytical framework, we can efficiently address and interpret the nonlinear connections within the data. To determine the most appropriate model for representing the connection between the TG/HDL‐C ratio and NAFLD, the log‐likelihood ratio was utilized.

To investigate subgroups on the basis of BMI, age, sex, DBP, SBP, drinking and smoking status, we categorized BMI (< 25, ≥ 25 kg/m^2^), age (< 60, ≥ 60 years), DBP (< 90, ≥ 90 mmHg), and SBP (< 140, ≥ 140 mmHg) on the basis of clinical cut points. A likelihood ratio test was used to assess the interactions between these subgroups, with *p*‐values of 0.05 or less deemed statistically significant.

## 3 Results

### 3.1 Basic Attributes of the Participants

In this study, we included 14,106 participants, with an average age of 43.51 ± 8.89 years, of whom 51.57% were male. Among the participants, 2403 were diagnosed with NAFLD. Table 1 provides a summary of the main demographic features, lab test outcomes, and other pertinent variables. The participants were divided into four groups according to their TG/HDL‐C ratio. The study found that those in the top quartile (Q4) had higher levels of age, WC, BMI, FPG, HBA1C, DBP, SBP, TC, TG, ALT, AST, GGT, and weight. Furthermore, the Q4 group exhibited a higher proportion of NAFLD patients, a greater number of males, and increased alcohol consumption and smoking. In contrast, participants in the lowest quartile (Q1) showed markedly higher HDL‐C levels than those in the other categories.

**TABLE 1:** The baseline characteristics of participants.

TG/HDLC quartile	Q1 (0.07–0.69)	Q2 (0.69–1.16)	Q3 (1.17–2.08)	Q4 (2.08–7.99)	*p*
Participants	3666	3593	3542	3305	
Age (years)	41.11 ± 8.32	43.15 ± 8.83	44.84 ± 9.10	45.15 ± 8.73	< 0.001
Sex					< 0.001
Female	2869 (78.26%)	2092 (58.22%)	1318 (37.21%)	552 (16.70%)	
Male	797 (21.74%)	1501 (41.78%)	2224 (62.79%)	2753 (83.30%)	
BMI (kg/m^2^)	20.28 ± 2.33	21.30 ± 2.63	22.53 ± 2.94	24.24 ± 3.11	< 0.001
Weight (kg)	53.11 ± 8.30	57.28 ± 9.77	62.04 ± 10.54	68.98 ± 11.18	< 0.001
WC (cm)	70.55 ± 6.81	73.72 ± 7.73	77.80 ± 8.16	83.11 ± 7.99	< 0.001
ALT (IU/L)	15.02 ± 7.69	16.82 ± 8.71	20.07 ± 11.41	27.34 ± 22.50	< 0.001
AST (IU/L)	16.97 ± 6.68	17.20 ± 5.92	18.17 ± 6.97	20.57 ± 13.14	< 0.001
GGT (IU/L)	13.77 ± 7.90	16.12 ± 11.96	20.27 ± 17.47	26.38 ± 20.85	< 0.001
HBA1C (%)	5.15 ± 0.29	5.15 ± 0.31	5.19 ± 0.33	5.22 ± 0.34	< 0.001
FPG (mg/dL)	89.42 ± 7.04	91.74 ± 7.22	93.84 ± 6.97	96.06 ± 6.67	< 0.001
SBP (mmHg)	108.12 ± 13.02	111.63 ± 14.08	115.90 ± 14.49	120.36 ± 14.71	< 0.001
DBP (mmHg)	66.80 ± 9.21	69.34 ± 9.78	72.59 ± 9.99	75.96 ± 10.13	< 0.001
TC (mg/dL)	188.20 ± 31.12	194.05 ± 31.54	200.14 ± 33.33	210.76 ± 33.97	< 0.001
HDL‐C (mg/dL)	70.91 ± 14.37	60.43 ± 11.25	51.90 ± 9.64	42.17 ± 7.82	< 0.001
TG (mg/dL)	33.90 ± 10.27	54.81 ± 11.43	79.82 ± 16.96	141.01 ± 45.36	< 0.001
Exercising status					0.002
Not‐regular exerciser	2981 (81.31%)	2969 (82.63%)	2911 (82.19%)	2800 (84.72%)	
Regular exerciser	685 (18.69%)	624 (17.37%)	631 (17.81%)	505 (15.28%)	
Smoking status					< 0.001
Non‐smokers	2958 (80.69%)	2457 (68.38%)	1914 (54.04%)	1366 (41.33%)	
Ex‐smoker	416 (11.35%)	582 (16.20%)	740 (20.89%)	794 (24.02%)	
Current smoker	292 (7.97%)	554 (15.42%)	888 (25.07%)	1145 (34.64%)	
NAFLD					< 0.001
No	3590 (97.93%)	3325 (92.54%)	2900 (81.87%)	1888 (57.13%)	
Yes	76 (2.07%)	268 (7.46%)	642 (18.13%)	1417 (42.87%)	
Alcohol consumption					< 0.001
Non‐consumer	3264 (89.03%)	3007 (83.69%)	2854 (80.58%)	2553 (77.25%)	
Light alcohol consumer	402 (10.97%)	586 (16.31%)	688 (19.42%)	752 (22.75%)	

Abbreviations: ALT, alanine aminotransferase; AST, aspartate aminotransferase; BMI, body mass index; DBP, diastolic blood pressure; FPG, fasting plasma glucose; GGT, gamma‐glutamyl transferase; HbA1c, glycosylated hemoglobin A1c, HDL‐C: high‐density lipoprotein cholesterol; NAFLD, Non‐alcoholic fatty liver disease; SBP, systolic blood pressure; TC, total cholesterol; TG, triglyceride; TG/HDL‐C, triglyceride‐to‐high‐density lipoprotein cholesterol ratio; WC, waist circumference.

### 3.2 The Results of Univariate Analysis

Table 2 displays the findings from the univariate analysis. The study found strong positive correlations between several factors and NAFLD, such as sex, age, BMI, WC, weight, DBP, SBP, AST, ALT, GGT, TC, TG, HbA1c, FPG, TG/HDL‐C ratio, and smoking. In contrast, HDL‐C levels showed a significant negative correlation with NAFLD risk. Moreover, participating in physical exercise was linked to a lower risk of NAFLD.

**TABLE 2:** The results of univariate analysis.

	Statistics	HR (95% CI)	*p*
TG/HDLC	1.56 ± 1.28	2.11 (2.03, 2.18)	< 0.001
Age (years)	43.51 ± 8.89	1.02 (1.01, 1.02)	< 0.001
Sex			
Female	6831 (48.43%)	1.0	
Male	7275 (51.57%)	4.85 (4.36, 5.40)	< 0.001
BMI (kg/m^2^)	22.03 ± 3.12	1.64 (1.61, 1.67)	< 0.001
Weight (kg)	60.13 ± 11.55	1.13 (1.12, 1.13)	< 0.001
WC (cm)	76.12 ± 8.98	1.20 (1.19, 1.21)	< 0.001
ALT (IU/L)	19.63 ± 14.40	1.10 (1.10, 1.11)	< 0.001
AST (IU/L)	18.17 ± 8.66	1.09 (1.08, 1.10)	< 0.001
GGT (IU/L)	18.95 ± 15.92	1.04 (1.04, 1.04)	< 0.001
HBA1C (%)	5.18 ± 0.32	4.43 (3.85, 5.11)	< 0.001
FPG (mg/dL)	92.67 ± 7.40	1.11 (1.11, 1.12)	< 0.001
SBP (mmHg)	113.83 ± 14.79	1.05 (1.05, 1.06)	< 0.001
DBP (mmHg)	71.05 ± 10.35	1.08 (1.07, 1.08)	< 0.001
TC (mg/dL)	197.97 ± 33.50	1.01 (1.01, 1.01)	< 0.001
HDL‐C (mg/dL)	56.73 ± 15.32	0.93 (0.92, 0.93)	< 0.001
TG (mg/dL)	75.85 ± 46.73	1.02 (1.02, 1.02)	< 0.001
Exercising status			
Not‐regular exerciser	11,661 (82.67%)	1.0	
Regular exerciser	2445 (17.33%)	0.82 (0.73, 0.93)	0.002
Smoking status			
Non‐smokers	8695 (61.64%)	1.0	
Ex‐smoker	2532 (17.95%)	2.11 (1.89, 2.35)	< 0.001
Current smoker	2879 (20.41%)	1.88 (1.69, 2.09)	< 0.001
Alcohol consumption			
Non‐consumer	11,678 (82.79%)	1.0	
Light alcohol consumer	2428 (17.21%)	0.97 (0.86, 1.09)	0.5683

Abbreviations: CI, confidence interval; Ref, reference.

### 3.3 The Relationship Between TG/HDL‐C Ratio and NAFLD

Table 3 illustrates the association between the TG/HDL‐C ratio and NAFLD across various models using Cox proportional hazards regression analysis. In Model 1, without any adjustments, the odds ratio (OR) for the TG/HDL‐C ratio with respect to NAFLD was 2.11 (95% CI: 2.03, 2.18). After minimal adjustment for sex and age in Model 2, the OR (95% CI) changed to 1.82 (1.81, 1.95). In Model 3, which was fully adjusted for factors such as sex, age, BMI, WC, weight, AST, ALT, GGT, SBP, DBP, HbA1c, FPG, TC, smoking, exercise, and drinking, the odds ratio (95% confidence interval) was 1.37 (1.31, 1.43). This indicates that for each unit increase in the TG/HDL‐C ratio, the risk of NAFLD increases by 37%.

**TABLE 3:** Relationship between the TG/HDL‐C ratio and NAFLD in different models.

Variables	Model 1 OR (95% CI) *p*	Model 2 OR (95% CI) *p*	Model 3 OR (95% CI) *p*
TG/HDLC	2.11 (2.03, 2.18) < 0.0001	1.88 (1.81, 1.95) < 0.0001	1.37 (1.31, 1.43) < 0.0001
*TG/HDLC quartile*			
Q1	1.0	1.0	1.0
Q2	3.81 (2.94, 4.93) < 0.0001	3.23 (2.48, 4.19) < 0.0001	1.95 (1.46, 2.60) < 0.0001
Q3	10.46 (8.20, 13.33) < 0.0001	7.65 (5.97, 9.80) < 0.0001	2.99 (2.28, 3.93) < 0.0001
Q4	35.45 (27.96, 44.95) < 0.0001	22.94 (17.93, 29.35) < 0.0001	5.20 (3.95, 6.84) < 0.0001
P for trend	< 0.001	< 0.001	< 0.001

*Note:* Model 1: we did not adjust for other covariants. Model 2: we adjusted for age and sex. Model 3: we adjusted for age, sex, BMI, WC, weight, ALT, AST, GGT, SBP, DBP, FPG, HbA1c, TC, exercising status, smoking status, and drinking status.

To verify the reliability of our outcomes, we carried out more sensitivity analyses. TG/HDL‐C was changed from a continuous to a categorical variable, and then it was re‐added to the model after being categorized. When TG/HDL‐C was treated as a categorical variable, we found a 420% increase in NAFLD risk for the highest tertiles compared to the lowest tertiles, with significant differences observed across tertiles (P for trend < 0.05), indicating a potential nonlinear correlation between the TG/HDL‐C ratio and NAFLD.

### 3.4 The Analysis of the Nonlinear Relationship

**FIGURE 2:**
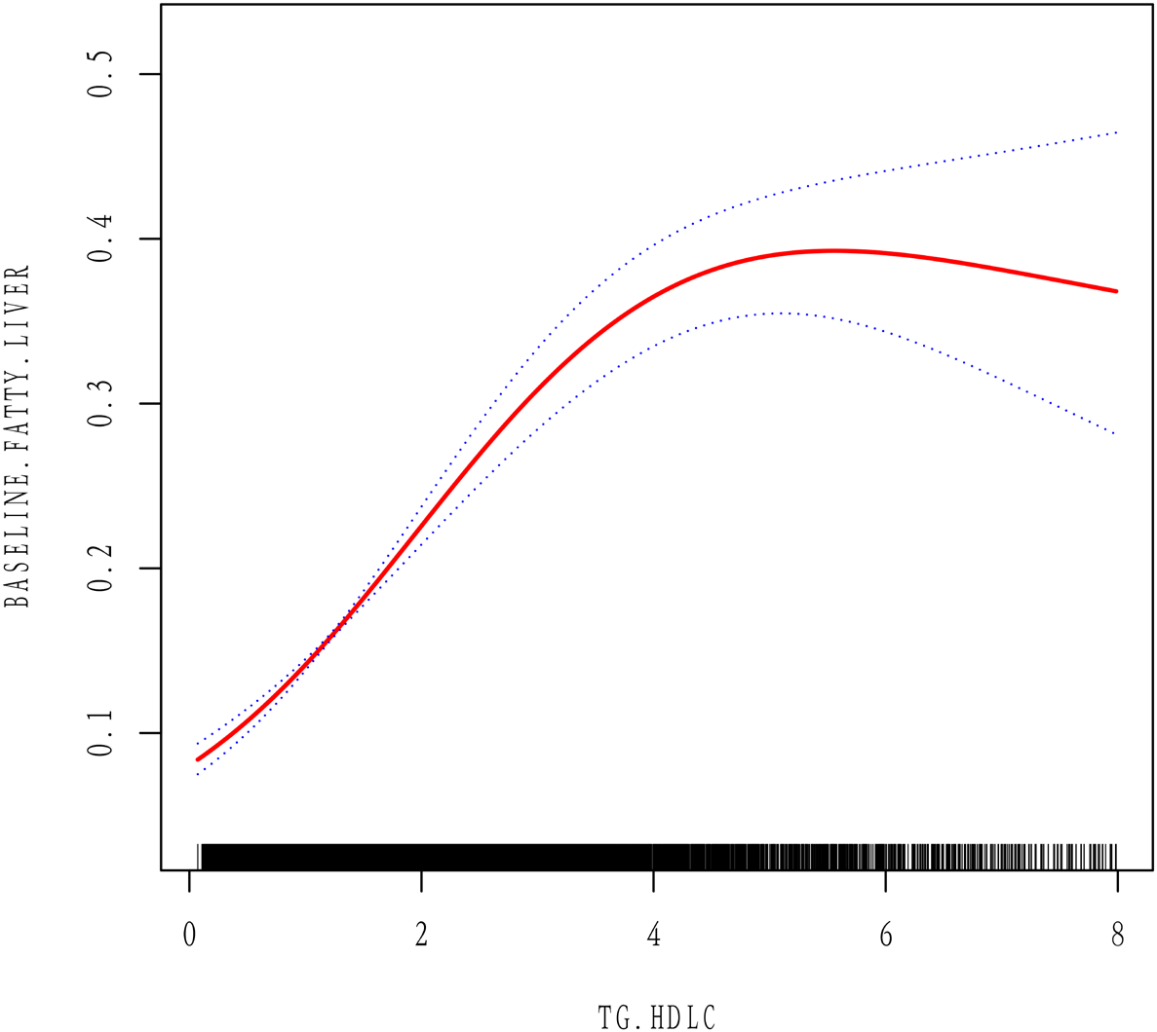
Association between TG/HDL‐C ratio and NAFLD. A threshold and non‐linear association between TG/HDL‐C ratio and NAFLD was found (*p* < 0.05) using a generalized additive model (GAM) after adjusting for sex, age, BMI, WC, weight,AST, ALT, GGT, SBP, DBP, HbA1c,FPG, TC, smoking, exercise, and drinking. The smooth curve fitting the variables is shown by a solid red line. The blue bands indicate the 95% confidence interval of the fit.

The curve fitting chart shows the nonlinear connection between the TG/HDL‐C ratio and NAFLD (Figure 2). The TG/HDL‐C ratio and NAFLD showed a nonlinear correlation after adjusting for confounding variables (Table 4). In the segmented Cox proportional hazards regression model, the TG/HDL‐C ratio reaches a turning point at 1.42 mmol/L (P for log‐likelihood ratio test < 0.001). To the left of this inflection point, the odds ratio (OR) is 3.71 (95% confidence interval: 2.87, 4.79), whereas it decreases to 1.23 (95% confidence interval: 1.17, 1.29) to the right.

**TABLE 4:** The result of the two‐piecewise linear regression model.

NAFLD	OR, 95% CI	*p*
Fitting model by standard linear regression	1.37 (1.31, 1.43)	< 0.001
*Fitting model by two‐piecewise linear regression*		
Infection point of TG/HDL‐C	1.42	
≤ 1.42	3.71 (2.87, 4.79)	< 0.001
> 1.42	1.23 (1.17, 1.29)	< 0.001
P for log likelihood ratio test	< 0.001	

*Note:* The nonlinear relationship between TG/HDL‐C and NAFLD. The nonlinear relationship between TG/HDL‐C and NAFLD was detected after adjusting for age, sex, BMI, WC, weight, ALT, AST, GGT, SBP, DBP, FPG, HbA1c, TC, exercising status, smoking status, and drinking status.

### 3.5 Subgroup Analysis

Subgroup analysis was conducted to explore other risk factors that may influence the relationship between the TG/HDL‐C ratio and NAFLD. We selected age, sex, smoking status, drinking status, DBP, SBP, and BMI as stratifying factors. Following that, we explored the effect size trends for these factors (Table 5). The results showed that light drinking, DBP, and SBP had no significant impact on the correlation between the TG/HDL‐C ratio and NAFLD risk. However, a stronger association between the TG/HDL‐C ratio and NAFLD risk was observed in individuals aged under 60, females, and those with a BMI of less than 25 kg/m^2^.

**TABLE 5:** Effect sizes of TG/HDL‐C ratio and NAFLD in subgroups.

Characteristic	No of patients	HR (95% CI)	*p*	P for interaction
BMI (kg/m^2^)				0.0102
≥ 5	2199	1.33 (1.23, 1.43)	< 0.0001	
< 5	11,907	1.39 (1.32, 1.47)	< 0.0001	
Sex				0.0027
Female	6831	1.63 (1.47, 1.81)	0.004	
Male	7275	1.32 (1.26, 1.39)	< 0.0001	
Age (years)				0.0208
≥ 60	625	1.16 (0.96, 1.39)	0.1265	
< 60	13,481	1.39 (1.33, 1.45)	< 0.0001	
Smoking status				0.0447
Non‐smokers	8695	1.46 (1.37, 1.57)	< 0.0001	
Ex‐smoker	2532	1.33 (1.22, 1.45)	< 0.0001	
Current smoker	2879	1.28 (1.19, 1.39)	< 0.0001	
Alcohol consumption				0.7357
Non‐consumer	11,678	1.27 (1.14, 1.40)	< 0.0001	
Light alcohol consumer	2428	1.52 (1.25, 1.85)	< 0.0001	
SBP (mmHg)				0.5181
≥ 140	641	1.53 (1.28, 1.82)	< 0.0001	
< 140	13,465	1.36 (1.30, 1.42)	< 0.0001	
DBP (mmHg)				0.9970
≥ 90	615	1.45 (1.23, 1.72)	< 0.0001	
< 90	13,491	1.37 (1.31, 1.43)	< 0.0001	

## 4 Discussion

Our retrospective study results indicate a significant correlation between the TG/HDL‐C ratio and NAFLD. After adjusting for other confounding factors, we found that for each one‐unit increase in TG/HDL‐C, the risk of NAFLD increased by 37%. Furthermore, we discovered that the relationship between TG/HDL‐C and NAFLD is nonlinear, particularly on either side of the inflection point. When TG/HDL‐C is less than or equal to 1.42, the positive correlation with NAFLD is more pronounced compared to the right side of the inflection point, where the correlation is weaker. Notably, in individuals younger than 60 years, females, and those with a BMI below 25 kg/m^2^, the association between TG/HDL‐C and NAFLD risk is stronger. These findings highlight the importance of TG/HDL‐C in assessing NAFLD risk, especially within specific populations.

The TG/HDL‐C ratio has emerged as a multifactorial biomarker with considerable significance across a spectrum of diseases, notably in relation to cardiovascular risk, metabolic syndrome, hypertension, diabetes, and chronic kidney disease. Studies have demonstrated that elevated triglyceride levels coupled with reduced HDL levels contribute to the progression of atherosclerosis, thereby highlighting the predictive capacity of the TG/HDL‐C ratio for coronary heart disease, especially among patients with diabetes and acute coronary syndrome [15]. Further research has identified the TG/HDL‐C ratio as a marker for metabolic syndrome and its components, reinforcing its status as a critical indicator of health risk within diabetic populations [16]. Additionally, empirical evidence has documented an 18% increase in the risk of developing hypertension for each standard deviation increase in the TG/HDL‐C ratio [17]. Moreover, the TG/HDL‐C ratio has implications for chronic kidney disease. Research conducted by Raikou et al. revealed significant associations between elevated TG/HDL‐C levels and decreased glomerular filtration rates, suggesting that this ratio may serve as a risk factor for CKD development in individuals without diabetes [18].

Earlier research has also distinctly shown a strong link between the TG/HDL‐C ratio and NAFLD. For instance, a cross‐sectional study with a substantial group of children and teenagers identified an independent link between TG/HDL‐C and NAFLD [19]. Similarly, a cohort study revealed that higher levels of TG/HDL‐C are closely related to increased risks of fatty liver and NAFLD [20]. Furthermore, a cross‐sectional study involving 18,061 patients found that, even after adjusting for factors such as FPG, TG, TC, LDL‐C, HDL‐C, UA, and Scrc, TG/HDL‐C remained independently associated with NAFLD risk. Specifically, the prevalence of NAFLD gradually increased among the higher quartiles of TG/HDL‐C (Q2–Q4), compared to the first quartile (Q1) [21]. Our current study also consistently reveals a close relationship between TG/HDL‐C and NAFLD, showing that after controlling for confounding variables, the prevalence and ORs of NAFLD also progressively rise within the quartiles of TG/HDL‐C.

Compared to earlier studies, we have made significant advancements in the resolution of nonlinearity. After accounting for possible confounding factors, we found that the inflection point of the TG/HDL‐C ratio is 1.42. Below this threshold, increasing the TG/HDL‐C ratio by one unit results in a 271% higher risk of developing NAFLD (OR = 3.71, 95% CI: 2.87, 4.79). Conversely, beyond this inflection point, every unit rise in the TG/HDL‐C ratio leads to just a 23% rise in risk (OR = 1.23, 95% CI: 1.17, 1.29). Additionally, we controlled for a wider array of biochemical indicators in our research, including BMI, WC, AST, ALT, TC, FPG, GGT, smoking, drinking, and exercise. A significant amount of evidence suggests that these parameters are related to the risk of NAFLD [22, 23, 24, 25, 26, 27]. By conducting various sensitivity analyses, including adjustments for target variables and subgroup analyses, we found stronger positive correlations in populations that are female, under 60 years of age, and with a BMI less than 25 kg/m^2^. These findings carry significant clinical implications.

We postulated the potential biological mechanisms linking the TG/HDL‐C ratio to NAFLD. Insulin resistance is positively associated with the TG/HDL‐C ratio [28]. Insulin resistance diminishes the sensitivity of adipose tissue to insulin, resulting in an increased release of free fatty acids (FFAs) from adipocytes and augmented TG synthesis within the liver. Furthermore, insulin resistance impairs the activity of lipoprotein lipase, thereby reducing the breakdown of TGs and elevating TG levels. Elevated TG levels exacerbate metabolic disturbances; if the liver's capacity to synthesize very low‐density lipoprotein (VLDL) is insufficient following excessive FFA influx, TG accumulation ensues, culminating in NAFLD [29, 30]. Moreover, high TG levels exacerbate insulin resistance in peripheral tissues, establishing a vicious cycle that accelerates the progression of NAFLD [30, 31, 32].

Simultaneously, the TG/HDL‐C ratio is correlated with chronic inflammatory responses, wherein hepatic inflammation may exacerbate the onset and progression of NAFLD [33, 34, 35, 36]. Chronic low‐grade inflammation is crucial in triggering fatty liver disease early on. A slightly elevated TG/HDL‐C ratio indicates systemic metabolic stress, with dysfunctional fat tissue releasing pro‐inflammatory substances like IL‐6, TNF‐α, and leptin, whereas HDL particles lose their anti‐inflammatory effects [37, 38]. This inflammation affects the liver through the portal circulation, activating Kupffer cells and inflammatory pathways in liver cells [39, 40]. This creates a pro‐inflammatory environment that advances NAFLD, even without significant liver fat accumulation [41].

Furthermore, estrogen significantly influences fat storage by enhancing lipoprotein lipase activity, increasing HDL‐C, reducing TG, and inhibiting VLDL secretion [42, 43]. Women, with higher estrogen levels, typically have lower TG/HDL‐C ratios, which help prevent NAFLD [43, 44]. Young women with a BMI under 25 often lack traditional risk factors like obesity, hypertension, or diabetes, so any metabolic markers like elevated TG/HDL‐C are crucial. In contrast, NAFLD in men, older adults, or those who are overweight is usually due to multiple factors, such as excess visceral fat and chronic disease, making the TG/HDL‐C ratio less significant.

In this study, the association between the TG/HDL‐C ratio and NAFLD weakens when the ratio exceeds 1.42, possibly because some individuals are receiving medication or lifestyle interventions. This reduced risk might reflect unmeasured confounding factors, such as lipid‐lowering treatments or lifestyle changes improving NAFLD [45, 46]. However, the study only uses baseline TG data and lacks details on treatments or behavioral changes. Future research should include longitudinal data on TG and HDL levels, as well as information on medication and lifestyle changes, to better understand these relationships. Nonetheless, the comprehensive results still reveal a positive correlation between TG/HDL‐C and NAFLD.

There are several notable strengths in our study. First, it further explores the positive nonlinear association between TG/HDL‐C and NAFLD, providing new insights for risk management of NAFLD. This finding not only enriches our understanding of the role of TG/HDL‐C in the development of NAFLD but also offers important reference points for clinical practice. Secondly, we performed sensitivity analyses to confirm the reliability of our results. These analyses included converting TG/HDL‐C into categorical variables and performing subgroup analyses, aiming to comprehensively explore the relationship between TG/HDL‐C and NAFLD. Such an approach aids in better understanding the impact of TG/HDL‐C across different populations. Third, the NAGALA project sample represents a large general health check population, which makes our study findings closely related to public health promotion and enhances their broader applicability and impact.

Nonetheless, recognizing the possible limitations of this study is essential. Firstly, the applicability of the findings may be limited because of the exclusive use of data from a Japanese cohort, which may restrict the generalizability of the conclusions to other racial or ethnic groups and populations outside of Japan. Additionally, although adjustments were made for a comprehensive set of covariates, including sex, age, BMI, WC, weight, AST, ALT, GGT, SBP, DBP, HbA1c, FPG, TC, smoking, exercise, and alcohol consumption, the possibility of potential confounding from unmeasured factors, such as medication use, dietary habits, or genetic predispositions, cannot be entirely excluded. Furthermore, the reliance on abdominal ultrasound for diagnosing non‐alcoholic fatty liver disease (NAFLD) may lead to an underestimation of hepatic fat content in certain individuals, thereby affecting the accuracy of the study's findings.

To conclude, the findings of this research show that the TG/HDL‐C ratio is an independent risk factor for NAFLD, even when other confounding factors are accounted for. Moreover, it shows a nonlinear relationship between the TG/HDL‐C ratio and the risk of NAFLD, with a particularly strong link when the ratio is under 1.42. These results imply that decreasing the TG/HDL‐C ratio could be a wise approach to lowering the risk of NAFLD.

## Author Contributions

Conceptualization and research design; L.S. and H.P. Supervision: Y.L. and H.P. Project administration: W.C. and H.P. Writing – Original draft preparation: L.S. and D.Z. Writing – Reviewing and Editing: H.P., L.S., Y.L, D.Z., and W.C. The author(s) read and approved the final manuscript.

## Ethics Statement

In the previously published article [13], Akuro Okamura et al. stated that the study was conducted according to the Declaration of Helsinki, and the Ethics Committee of Murakami Memorial Hospital approved the original research.

## Consent

The authors have nothing to report.

## Conflicts of Interest

The authors declare no conflicts of interest.

## Data Availability

The raw data can be downloaded from the ‘DATADRYAD’ database (www.Datadryad.org). Dryad Digital Repository. https://doi.org/10.5061/dryad.8q0p192.

## References

[1] M. Yang , J. Rigdon , and S. Tsai , “Association of Triglyceride to HDL Cholesterol Ratio With Cardiometabolic Outcomes,” Journal of Investigative Medicine 67, no. 3 (2019): 663–668. 30530527 10.1136/jim-2018-000869

[2] B. Dong , Y. Mao , Z. Li , and F. Yu , “The Value of the Atherogenic Index of Plasma in Non‐Obese People With Non‐Alcoholic Fatty Liver Disease: A Secondary Analysis Based on a Cross‐Sectional Study,” Lipids in Health and Disease 19, no. 1 (2020): 148. 32576204 10.1186/s12944-020-01319-2 PMC7313140

[3] M. Hung , C. F. Chen , M. Tsou , H. Lin , L. Hwang , and C. H. Hsu , “Relationship Between Gallstone Disease and Cardiometabolic Risk Factors in Elderly People With Non‐Alcoholic Fatty Liver Disease,” Diabetes Metabolic Syndrome and Obesity Targets and Therapy 13 (2020): 3579–3585. 33116709 10.2147/DMSO.S266947 PMC7553650

[4] T. Onay and A. Uçar , “Which Anthropometric Measurement/Ratio Is a Better Predictor of Non‐Alcoholic Fatty Liver Disease?,” (2023).

[5] G. Wen , P. Qin , X. Li , et al., “Correlation of the Lipid Ratios With Hepatic Steatosis and Liver Fibrosis in Non‐Alcoholic Fatty Liver Disease Patients,” (2020).

[6] Y. Zou , L. Zhong , C. Hu , M. Zhong , N. Peng , and G. Sheng , “LDL/HDL Cholesterol Ratio Is Associated With New‐Onset NAFLD in Chinese Non‐Obese People With Normal Lipids: A 5‐Year Longitudinal Cohort Study,” Lipids in Health and Disease 20, no. 1 (2021): 28. 33766067 10.1186/s12944-021-01457-1 PMC7993485

[7] Q. Wang , D. Zheng , J. Liu , F. Li , and L. X. Qiu , “Atherogenic Index of Plasma Is a Novel Predictor of Non‐Alcoholic Fatty Liver Disease in Obese Participants: A Cross‐Sectional Study,” Lipids in Health and Disease 17, no. 1 (2018): 284. 30545385 10.1186/s12944-018-0932-0 PMC6293612

[8] I. C. Efrem , M. Moța , I. M. Vladu , et al., “A Study of Biomarkers Associated With Metabolic Dysfunction‐Associated Fatty Liver Disease in Patients With Type 2 Diabetes,” Diagnostics 12, no. 10 (2022): 2426. 36292115 10.3390/diagnostics12102426 PMC9600788

[9] Z. Chen , H. Qin , S. Qiu , G. Chen , and Y. Chen , “Correlation of Triglyceride to High‐Density Lipoprotein Cholesterol Ratio With Nonalcoholic Fatty Liver Disease Among the Non‐Obese Chinese Population With Normal Blood Lipid Levels: A Retrospective Cohort Research,” Lipids in Health and Disease 18, no. 1 (2019): 162. 31399032 10.1186/s12944-019-1104-6 PMC6689160

[10] F. Gao , H. Li , X. Wang , Y. Zheng , X. Yang , and J. Ren , “Comparison of Triglyceride/HDL‐C Ratio and Triglyceride Glucose Index in Identifying NAFLD in Chinese Population: A Cross‐Sectional Study,” (2021).

[11] H. Tutunchi , F. Naeini , M. Ebrahimi‐Mameghani , M. Mobasseri , S. Naghshi , and A. Ostadrahimi , “The Association of the Steatosis Severity, NAFLD Fibrosis Score and FIB‐4 Index With Atherogenic Dyslipidaemia in Adult Patients With NAFLD: A Cross‐Sectional Study,” International Journal of Clinical Practice 75, no. 6 (2021): e14131. 33683797 10.1111/ijcp.14131

[12] G. Sheng , S. Lu , Q. Xie , N. Peng , M. Kuang , and Y. Zou , “The Usefulness of Obesity and Lipid‐Related Indices to Predict the Presence of Non‐Alcoholic Fatty Liver Disease,” Lipids in Health and Disease 20, no. 1 (2021): 134. 34629059 10.1186/s12944-021-01561-2 PMC8502416

[13] T. Okamura , Y. Hashimoto , M. Hamaguchi , A. Obora , T. Kojima , and M. Fukui , “Ectopic Fat Obesity Presents the Greatest Risk for Incident Type 2 Diabetes: A Population‐Based Longitudinal Study,” International Journal of Obesity 43, no. 1 (2019): 139–148. 29717276 10.1038/s41366-018-0076-3

[14] M. Hamaguchi , T. Kojima , Y. Itoh , et al., “The Severity of Ultrasonographic Findings in Nonalcoholic Fatty Liver Disease Reflects the Metabolic Syndrome and Visceral Fat Accumulation,” American Journal of Gastroenterology 102, no. 12 (2007): 2708–2715. 17894848 10.1111/j.1572-0241.2007.01526.x

[15] D. Shi , L. Wang , and H. Cong , “Association Between Triglycerides to High‐Density Lipoprotein Cholesterol Ratio and Death Risk in Diabetic Patients With New‐Onset Acute Coronary Syndrome: A Retrospective Cohort Study in the Han Chinese Population,” Reviews in Cardiovascular Medicine 23, no. 6 (2022): 190. 39077180 10.31083/j.rcm2306190 PMC11273665

[16] M. R. Azarpazhooh , F. Najafi , M. Darbandi , S. Kiarasi , T. Oduyemi , and J. D. Spence , “Triglyceride/High‐Density Lipoprotein Cholesterol Ratio: A Clue to Metabolic Syndrome, Insulin Resistance, and Severe Atherosclerosis,” Lipids 56, no. 4 (2021): 405–412. 33881177 10.1002/lipd.12302

[17] M. Tohidi , M. Hatami , F. Hadaegh , and F. Azizi , “Triglycerides and Triglycerides to High‐Density Lipoprotein Cholesterol Ratio Are Strong Predictors of Incident Hypertension in Middle Eastern Women,” Journal of Human Hypertension 26, no. 9 (2012): 525–532. 21776016 10.1038/jhh.2011.70

[18] V. D. Raikou , D. Kyriaki , and S. Gavriil , “Triglycerides to High‐Density Lipoprotein Cholesterol Ratio Predicts Chronic Renal Disease in Patients Without Diabetes Mellitus (STELLA Study),” Journal of Cardiovascular Development and Disease 7, no. 3 (2020): 28. 32752179 10.3390/jcdd7030028 PMC7570173

[19] L. Pacifico , E. Bonci , G. Andreoli , et al., “Association of Serum Triglyceride‐To‐HDL Cholesterol Ratio With Carotid Artery Intima‐Media Thickness, Insulin Resistance and Nonalcoholic Fatty Liver Disease in Children and Adolescents,” Nutrition, Metabolism, and Cardiovascular Diseases 24, no. 7 (2014): 737–743. 10.1016/j.numecd.2014.01.010 24656140

[20] Y. Fukuda , Y. Hashimoto , M. Hamaguchi , et al., “Triglycerides to High‐Density Lipoprotein Cholesterol Ratio Is an Independent Predictor of Incident Fatty Liver; a Population‐Based Cohort Study,” Liver International 36, no. 5 (2016): 713–720. 26444696 10.1111/liv.12977

[21] N. Fan , L. Peng , Z. Xia , et al., “Triglycerides to High‐Density Lipoprotein Cholesterol Ratio as a Surrogate for Nonalcoholic Fatty Liver Disease: A Cross‐Sectional Study,” Lipids in Health and Disease 18, no. 1 (2019): 39. 30711017 10.1186/s12944-019-0986-7 PMC6359827

[22] Q. Zhou , Y. Wang , J. Wang , et al., “Prevalence and Risk Factor Analysis for the Nonalcoholic Fatty Liver Disease in Patients With Type 2 Diabetes Mellitus,” Medicine 100, no. 10 (2021): e24940. 33725855 10.1097/MD.0000000000024940 PMC7969325

[23] P. Lü , S. Wu , N. Zhou , S. Zhu , Q. Liu , and X. Li , “Clinical Characteristics and Risk Factors of Nonalcoholic Fatty Liver Disease in Children With Obesity,” BMC Pediatrics 21, no. 1 (2021): 122. 33711964 10.1186/s12887-021-02595-2 PMC7953770

[24] V. Jain , M. Jana , B. Upadhyay , et al., “Prevalence, Clinical & Biochemical Correlates of Non‐Alcoholic Fatty Liver Disease in Overweight Adolescents,” Indian Journal of Medical Research 148, no. 3 (2018): 291. 30425219 10.4103/ijmr.IJMR_1966_16 PMC6251268

[25] M. Lahelma , P. K. Luukkonen , S. U. Qadri , et al., “Assessment of Lifestyle Factors Helps to Identify Liver Fibrosis due to Non‐Alcoholic Fatty Liver Disease in Obesity,” Nutrients 13, no. 1 (2021): 169. 33429859 10.3390/nu13010169 PMC7827136

[26] Z. Hoseini , N. Behpoor , and R. Hoseini , “The Association of Physical Activity Level and the Risk Factors of Nonalcoholic Fatty Liver Disease in the Elderly Female Patients,” Journal of Kermanshah University of Medical Sciences 24, no. 3 (2020): e108085.

[27] Z. Zhang , J. Wang , and H. Wang , “Correlation of Blood Glucose, Serum Chemerin and Insulin Resistance With NAFLD in Patients With Type 2 Diabetes Mellitus,” Experimental and Therapeutic Medicine 15 (2018): 2936–2940. 29456698 10.3892/etm.2018.5753 PMC5795563

[28] R. Gong , G. Luo , M. Wang , L. Ma , S. Sun , and X. Wei , “Associations Between TG/HDL Ratio and Insulin Resistance in the US Population: A Cross‐Sectional Study,” Endocrine Connections 10, no. 11 (2021): 1502–1512. 34678755 10.1530/EC-21-0414 PMC8630769

[29] M. Alcala , M. Calderon‐Dominguez , D. Serra , L. Herrero , M. P. Ramos , and M. Viana , “Short‐Term Vitamin E Treatment Impairs Reactive Oxygen Species Signaling Required for Adipose Tissue Expansion, Resulting in Fatty Liver and Insulin Resistance in Obese Mice,” PLoS One 12, no. 10 (2017): e0186579. 29028831 10.1371/journal.pone.0186579 PMC5640231

[30] F. Benhamed , P. Denechaud , M. Lemoine , et al., “The Lipogenic Transcription Factor ChREBP Dissociates Hepatic Steatosis From Insulin Resistance in Mice and Humans,” Journal of Clinical Investigation 122, no. 6 (2012): 2176–2194. 22546860 10.1172/JCI41636 PMC3366390

[31] H. Kitade , G. Chen , and T. Ota , “Nonalcoholic Fatty Liver Disease and Insulin Resistance: New Insights and Potential New Treatments,” Nutrients 9, no. 4 (2017): 387. 28420094 10.3390/nu9040387 PMC5409726

[32] W. C. Yeh , Y. C. Tsao , W. C. Li , I. S. Tzeng , L. S. Chen , and J. Y. Chen , “Elevated Triglyceride‐To‐HDL Cholesterol Ratio Is an Indicator for Insulin Resistance in Middle‐Aged and Elderly Taiwanese Population: A Cross‐Sectional Study,” Lipids in Health and Disease 18, no. 1 (2019): 176. 31604438 10.1186/s12944-019-1123-3 PMC6790048

[33] S. A. M. Bahnasawy , N. E. S. El Gammal , N. I. El Attar , and A. M. El‐Gebaly , “Liver Fatty Acid‐Binding Protein (L‐Fabp) as a Diagnostic Marker for Non‐Alcoholic Fatty Liver Disease,” Egyptian Journal of Hospital Medicine 91, no. 1 (2023): 5345–5352.

[34] E. Cobbina and F. Akhlaghi , “Non‐Alcoholic Fatty Liver Disease (NAFLD) – Pathogenesis, Classification, and Effect on Drug Metabolizing Enzymes and Transporters,” Drug Metabolism Reviews 49, no. 2 (2017): 197–211. 28303724 10.1080/03602532.2017.1293683 PMC5576152

[35] C. C. Wu , Y. Cheng , K. Chen , and C. T. Chien , “Deep Sea Water‐Dissolved Organic Matter Intake Improves Hyperlipidemia and Inhibits Thrombus Formation and Vascular Inflammation in High‐Fat Diet Hamsters,” Life 12, no. 1 (2022): 82. 35054478 10.3390/life12010082 PMC8778340

[36] M. Amir , M. Yu , P. He , and S. Srinivasan , “Hepatic Autonomic Nervous System and Neurotrophic Factors Regulate the Pathogenesis and Progression of Non‐Alcoholic Fatty Liver Disease,” Frontiers in Medicine 7 (2020): 62. 32175323 10.3389/fmed.2020.00062 PMC7056867

[37] J. Grisouard , E. Bouillet , K. Timper , et al., “Both Inflammatory and Classical Lipolytic Pathways Are Involved in Lipopolysaccharide‐Induced Lipolysis in Human Adipocytes,” Innate Immunity 18, no. 1 (2010): 25–34. 21088047 10.1177/1753425910386632

[38] N. S. Kalupahana , K. Claycombe , and N. Moustaïd Moussa , “(N‐3) Fatty Acids Alleviate Adipose Tissue Inflammation and Insulin Resistance: Mechanistic Insights,” Advances in Nutrition 2, no. 4 (2011): 304–316. 22332072 10.3945/an.111.000505 PMC3125680

[39] R. P. Santiago , C. V. B. Figueiredo , L. M. Fiuza , et al., “Transforming Growth Factor Beta Receptor 3 Haplotypes in Sickle Cell Disease Are Associated With Lipid Profile and Clinical Manifestations,” Mediators of Inflammation 2020 (2020): 1–18. 10.1155/2020/3185015 PMC7603616 33149723

[40] Y. M. Yang and E. Seki , “TNFα in Liver Fibrosis,” Current Pathobiology Reports 3, no. 4 (2015): 253–261. 26726307 10.1007/s40139-015-0093-z PMC4693602

[41] S. N. Gregory , S. R. Perati , and Z. J. Brown , “Alteration in Immune Function in Patients With Fatty Liver Disease,” Hepatoma Research 8 (2022): 31.

[42] A. P. Frank , S. R. de Souza , B. F. Palmer , and D. J. Clegg , “Determinants of Body Fat Distribution in Humans May Provide Insight About Obesity‐Related Health Risks,” Journal of Lipid Research 60, no. 10 (2019): 1710–1719. 30097511 10.1194/jlr.R086975 PMC6795075

[43] S. E. Kim , J. Min , S. Lee , D. Y. Lee , and D. Choi , “Different Effects of Menopausal Hormone Therapy on Non‐Alcoholic Fatty Liver Disease Based on the Route of Estrogen Administration,” Scientific Reports 13 (2023): 15461. 37726372 10.1038/s41598-023-42788-6 PMC10509271

[44] H. Yang , G. Chen , M. Zhou , et al., “Association of Age at First Birth and Risk of Non‐Alcoholic Fatty Liver Disease in Women: Evidence From the NHANES,” Hepatology International 17, no. 2 (2022): 303–312. 36227515 10.1007/s12072-022-10429-1

[45] P. Charatcharoenwitthaya , K. Kuljiratitikal , O. Aksornchanya , K. Chaiyasoot , W. Bandidniyamanon , and N. Charatcharoenwitthaya , “Moderate‐Intensity Aerobic vs Resistance Exercise and Dietary Modification in Patients With Nonalcoholic Fatty Liver Disease: A Randomized Clinical Trial,” Clinical and Translational Gastroenterology 12, no. 3 (2021): e00316. 33939383 10.14309/ctg.0000000000000316 PMC7925136

[46] D. Zhang , S. Niu , Y. Ma , et al., “Fenofibrate Improves Insulin Resistance and Hepatic Steatosis and Regulates the Let‐7/SERCA2b Axis in High‐Fat Diet‐Induced Non‐Alcoholic Fatty Liver Disease Mice,” Frontiers in Pharmacology 12 (2021): 770652. 35126113 10.3389/fphar.2021.770652 PMC8807641

